# Progress in Neurology

**Published:** 1902-05-17

**Authors:** 


### PROGRESS IN NEUROLOGY.

**Cerebral Cortex.**—Sherrington and Grünbaum [1] have made important researches on the "motor" area of the higher apes—the chimpanzee, orang, and gorilla. Their stimulative experiments were made by a unipolar method, *i.e.* one electrode was applied to the cortex, and the other to one of the fore limbs. They found that no appreciable difference exists between the excitability of the cortex in the higher and lower monkeys. No movement resulted from stimulation of the ascending parietal convolution; all the motor areas are in front of the fissure of Rolando, and mainly confined to the ascending frontal gyrus, and the surface of this convolution which looks into the fissure of Rolando was also excitable. The order in which the areas are arranged from below upwards was (1) face and head, the former extending slightly on to the inferior frontal convolution, (2) neck, (3) shoulder, (4) arm, (5) upper part of trunk, (6) lower part of trunk, and (7) lower limb. The last-named area was small and confined to the sagittal face of the ascending frontal convolution. The order corresponded, therefore, exactly with the position of parts of the body. Removal of the arm or leg area was followed by a monoplegia, which disappeared in about five weeks. On the other hand removal of portions of cortex of the ascending parietal convolution were never followed by paralysis. The pyramidal degeneration in the case of the leg area involved the crossed pyramidal tract only, in the case of the arm there was also degeneration in the direct pyramidal tract on the same side. They could not find inexcitable areas between the various excitable ones, as has been affirmed by Beevor and Horsley. This investigation will prove of great service to surgeons operating on the human brain, especially the discovery of the inexcitability of the ascending parietal convolution.

**Muscular Tonus and the Reflexes.**—Interesting comparisons have been made by Crocq[2] between man and lower vertebrates of the degree to which the functions of the spinal cord are under the control of the brain. Muscular tonus is the resultant of two factors—the inherent elasticity of muscular tissue, and the permanent excitation supplied by the nervous centres. Now, if the cervical cord be cut across in the frog no diminution of muscular tonus follows ; in the dog or rabbit the tonus of the voluntary muscles is diminished and that of the sphincters increased, and these results are more marked in the monkey, whilst in man the exaggeration of sphincter tonus is accompanied by permanent and complete loss of that of the voluntary muscles. Thus, as the animal scale is ascended voluntary muscle tonus is more and more influenced by impulses proceeding from the brain. Sphincter tonus, on the other hand, is in all animals dependent solely on less remote impulses. Reflexes, also, are subserved by fibre systems which increase in length and complexity as the animal ascends the scale of evolution. The reflex troubles following injury to the cerebrum and upper part of the spinal cord are in the animal series proportional in extent to the development of the pyramidal tract.

**Action of Nicotine on Nerve Cells.**—Langley[3] found, some years ago, that the application of nicotine to a sympathetic ganglion prevented the passage of nerve impulses through it, while a similar application to the nerve trunk had no such hindering effect. He now reports further experiments on the subject. If the preganglionic nerves of a ganglion be cut, and time for degeneration allowed, the application of nicotine to the ganglion produced normal stimulating effects which must be due to direct action on the nerve cells. The alkaloid, however, had no stimulating effect on posterior root ganglia, nor did it paralyse the nerve cells of those bodies. On the other hand nicotine was a powerful stimulant to the nerve cells of the spinal cord, producing violent muscular twitchings in the muscles supplied from the region experimented upon, but, unlike what obtains in sympathetic ganglia, it did not stop the passage of nerve impulses through them. Thus there is a great difference in the behaviour to nicotine of various classes of neurons.

**Ankle Clonus.**—MacWilliam[4] records the result of observations on the rate of ankle clonus, which previous investigators had stated to be from six to nine per second. He found that if the recording tambour was strapped to the calf-muscle the rate in some cases was as high as 14 a second, and in these cases the rate as recorded by the movement of the foot was only half as great, seven per second. Thus, probably, there may be a fusion of two vibrations into one large curve, and this may be indicated by a notch of the top of the curves recorded by the foot. This is of interest in view of the statements of Schäfer and Horsley that the automatic action of the nerve cells of the spinal cord appears to be never capable of originating a rhythm of greater frequency than 10 per second.

[1] Lancet, Oct. 12, 1901. [2] Brit. Med. Jour., Jan. 25, 1902. [3] Brit. Med. Jour., Oct. 26, 1901. [4] Brit. Med. Jour., Nov. 30, 1901.

